# Suitability Evaluation of LaNi_5_ as Hydrogen-Storage-Alloy Actuator by In-Situ Displacement Measurement during Hydrogen Pressure Change

**DOI:** 10.3390/molecules24132420

**Published:** 2019-07-01

**Authors:** Kenta Goto, Tomoyuki Hirata, Isao Yamamoto, Wataru Nakao

**Affiliations:** 1 Graduate School of Engineering, Yokohama National University, 79-5 Tokiwadai, Hodogaya, Yokohama, Kanagawa 240-8501, Japan; 2 Faculty of Engineering, Yokohama National University, 79-5 Tokiwadai, Hodogaya, Yokohama, Kanagawa 240-8501, Japan

**Keywords:** metal hydride, lanthanum-nickel alloy, reaction kinetics, diffusion, lattice expansion

## Abstract

The swelling ability of LaNi_5_ for application to hydrogen-storage-alloy (HSA) actuator is discussed through the measurement of the swelling ratio in hydrogen. The HSA actuator is driven by hydrogen pressure change causing the swelling of HSA. LaNi_5_ is one of the candidate materials for HSA actuators as well as palladium. Some prototypes of HSA actuators using LaNi_5_ have been fabricated; however, the kinetic swelling ability of LaNi_5_ itself has been not investigated. In this paper, the authors investigated the static and kinetic swelling ability of LaNi_5_ powder under hydrogen atmosphere. The results showed that the swelling ratio increased by 0.12 at the phase transition pressure. Response time decreased with an increase in the charged pressure during absorption, while it remained constant during discharge. Reaction kinetics revealed that these swelling behaviors were explained by hydrogen absorption and lattice expansion. The swelling ability of LaNi_5_ was also compared with that of palladium. The results show that LaNi_5_ swells 1.8 times more than palladium under 0.5 MPa. LaNi_5_ is suitable for an actuator driven repeatedly under more than the phase transition pressure. Palladium can be used for one-way-operation actuator even under 0.1 MPa since its response time during the evacuation was much longer than during the pressurization.

## 1. Introduction

A hydrogen storage alloy (HSA) has a characteristic that its volume increases with absorbing hydrogen. The swelling is undesirable for applications such as hydrogen storage [1], rechargeable batteries [2], and heat pumps [3] since it causes pulverization and deformation of a tank surrounding the hydrogen storage alloy. Therefore, hydrogen storage alloys for these applications are designed to have a small swelling ratio.

A hydrogen-storage-alloy actuator uses the swelling characteristic. When hydrogen gas is charged into the actuator, it generates displacement and force induced by the swelling of hydrogen storage alloy. Until now, unimorph-shape [4,5,6,7,8,9,10] and capsule-shape [11,12] actuators have been developed using different alloys: palladium-nickel [4,5,6,7,12], lanthanum-nickel [8,9,10], and vanadium-titanium systems [7]. They consist of an HSA foil and a substrate with no hydrogen storage ability, and successfully showed repeatable actuation by changing hydrogen pressure.

It is important to evaluate the suitability of a hydrogen storage alloy for the actuator application. The requirements for an HSA employed in actuators used under the atmospheric pressure were discussed by Nakai et al. [4]: (1) phase transition pressure near the atmospheric pressure, (2) a large hydrogen content absorbed at the phase transition pressure, (3) a small hysteresis between the phase transition pressures at absorption and discharge, (4) a rapid hydrogen absorption and discharge, and (5) high pulverization resistance and good formability. The requirements (1)–(4) are referred in terms of the reactivity with hydrogen atoms. It is important for HSA to have the high pulverization resistance, because it cannot transfer stress induced by the swelling to the actuated material if the pulverization occurs. The materials in which plastic deformation easily occurs generally show high pulverization resistance as well as good formability. These requirements are critical because the performance of an HSA actuator is influenced by the hydrogen absorption/discharge characteristics of HSA; however, the swelling ability of HSA is not included in the requirements. 

The macroscopic volume change of HSA powder has been investigated, through the deformation of a sample holder [13,14,15], or directly by optical [16,17] and electrical [18,19] techniques. Most of them focused on the static performance from the viewpoints of the effect on a hydrogen tank. Goto et al. [16] investigated the kinetic absorption behavior of palladium powder, and revealed that diffusion of hydrogen atoms is dominant as a rate-determining step during the swelling. The information on these static and kinetic swelling characteristics is helpful to select a suitable hydrogen storage alloy in the design of an HSA actuator. 

LaNi_5_ alloy has been also used in the previous HSA actuators [8,9,10]. It has a high hydrogen absorption ability through the phase transition to LaNi_5_H_6_ around 0.2 MPa at room temperature [20]. It shows a high absorption and discharge rate and is easily activated at room temperature [21]. These characteristics of LaNi_5_ are attractive for an HSA actuator. Material parameters related to hydrogen absorption are tabulated in Table 1.

In this paper, the authors investigated the static and kinetic swelling behavior of LaNi_5_ powder during hydrogen absorption to evaluate its suitability as an actuator material. The variation of the swelling ratio with time was measured under hydrogen atmosphere at various pressures. The obtained characteristics were compared with those of palladium.

## 2. Results and Discussions

### 2.1. Static Properties—Equilibrium Swelling Ratio

**Figure 1:**
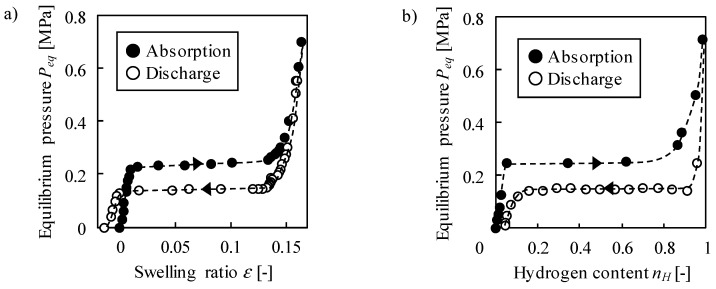
Variation of (**a**) swelling ratio and (**b**) hydrogen content with equilibrium pressure.

Figure 1a shows the change in swelling ratio *ε* with equilibrium hydrogen pressure *P_eq_*. During the pressurization, *ε* increased with *P_eq_*, where it drastically increased by 0.12 in the range of 0.220–0.254 MPa. *ε* was 0.17 at the maximum pressure of 0.698 MPa. During the depressurization, *P_eq_* was lower than that during the pressurization at the same *ε*. *ε* decreased by 0.13 at *P_eq_* = 0.130–0.155 MPa. Note that the measured values include free spaces, and the possible porosity change was not taken into account. Ribeiro and Gil [18] investigated the porosity during the swelling of LaNi_5_ powder through the measurement of electrical resistance, showing that it decreased by ~1% under 11 MPa. The negative value below 0.118 MPa during the discharge was caused by the porosity change. The same tendency was observed in the literature [16,17,19], in particular, the volume of LaNi_5_ continued to decrease at least up to 60 cycles, though the diameter was constant after 15 cycles in [16]. 

The dependence of the swelling ratio on the pressure agreed with that of the hydrogen content *n_H_* as shown in Figure 1b, where the unit of *n_H_* is a molar ratio of hydrogen to metal, H/M (= 1 at LaNi_5_H_6_). The phase transition pressures between solid solution (LaNi_5_) and hydride (LaNi_5_H_6_) were approximately 0.25 and 0.15 MPa during the absorption and discharge, respectively. The phase transition pressures almost agree with the plateau pressures at which *ε* dramatically changed, which is consistent with the previous works showing that the swelling of LaNi_5_ was induced by the absorbed hydrogen [25]. The discrepancy between the phase transition pressures in the swelling-ratio and hydrogen-content measurements were caused by the temperature mismatch because the phase transition pressure is sensitive to temperature, for example, it is 0.03 MPa higher at 25 ℃ than at 20 ℃ during discharge, which is estimated from the reaction enthalpy and entropy [26]. The lattice volume increases in proportion to the hydrogen content (See Appendix A). The relationship is represented as 20.7*n_H_* + 87.0 Å^3^, which corresponds to the lattice swelling ratio of 0.238. It is expected that *ε* shows a linear relationship with *n_H_* as well as the lattice volume if the temperature is precisely regulated and the porosity change is taken into account. 

### 2.2. Kinetic Properties—Response Time

**Figure 2:**
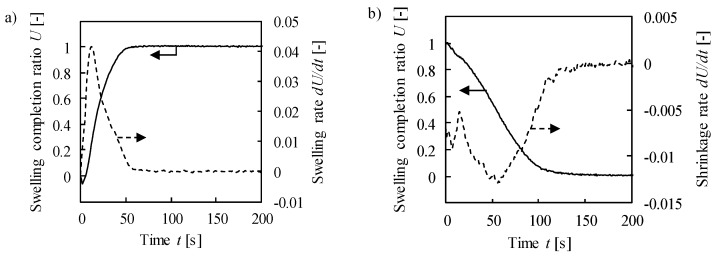
Time change of swelling completion ratio and its rate during (**a**) absorption under 0.518 MPa and (**b**) following discharge.

Figure 2 shows the change of swelling completion ratio *U* and its rate *dU*/*dt* with time *t* at the charged pressure *P_up_* = 0.518 MPa. The swelling completion ratio was calculated by dividing the swelling ratio at each time by the final swelling ratio at *t* = 300 s. For example, the final swelling ratio was 0.145 at *P_up_* = 0.518 MPa, which agrees with the equilibrium swelling ratio discussed in Section 2.1. The response rate *dU*/*dt* was defined as a slope of *U* with respect to time. The displacement decreased slightly soon after the pressurization due to the compression by hydrogen gas, and turned to raise at *t* = 2 s (Figure 2a). *dU*/*dt* reached a peak of 0.041 s^−1^ at 12 s and decreased. The response time at *U* = 0.8 (*t*_0.8_) was 33 s. A similar tendency was observed at the time of evacuation (Figure 2b), where the peak of *dU*/*dt* was –0.013 s^−1^ at 56 s, and *t_0.8_* was 82 s. The measurements at different *P_up_* showed a similar tendency.

**Figure 3:**
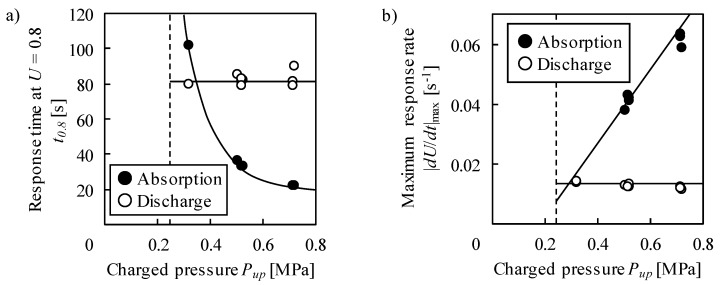
Dependence of (**a**) response time at *U* = 0.8 and (**b**) maximum response rate on pressure. Dashed lines represent the phase transition pressure during absorption.

The dependence of *t_0.8_* on *P_up_* was different during the absorption and discharge as shown in Figure 3a. The response time decreased with the increase in *P_up_* at the absorption process. It approaches infinity at a phase transition pressure of 0.25 MPa. During the discharge process, *t_0.8_* was constant. Figure 3b shows the change in the maximum absolute value of *dU*/*dt*, |*dU*/*dt*|_max_ with *P_up_*. |*dU*/*dt*|_max_ increased in proportion to *P_up_* in the absorption process, while it was constant in the discharge process. These tendencies are explained by the driving force of hydrogen absorption/discharge. The driving force of a reaction is decided by the difference between the applied pressure *P_ap_* and equilibrium pressure at the moment *P_e_*, i.e., |*P_ap_* − *P_e_*|. Here, *P_ap_* = *P_up_* during the absorption and *P_ap_* = 0 during the discharge. By extrapolating the linear relationship between *P_up_* and |*dU*/*dt*|_max_, |*dU*/*dt*|_max_ becomes almost 0 at the phase transition pressure in Figure 3b, which implies *P_e_* corresponds to the phase transition pressure. During the discharge, *P_e_* should also be constant so as to satisfy the constant *P_ap_* and |*dU*/*dt*|_max_; it can be the phase transition pressure. 

**Figure 4:**
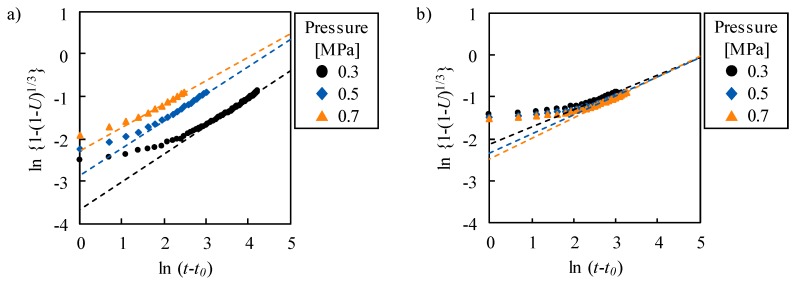
Relationship between swelling/shrinkage completion ratio and time during (**a**) absorption and (**b**) discharge. The dashed lines represent approximate straight lines at *U* > 0.5.

The rate-limiting step is determined from the analysis using reaction kinetics. The relationship between reaction rate *R* and time *t* is formulated as Equation (1) in the unreacted-core model [27].

(1) $$\ln\left\{1-{\left(1-R\right)}^{\frac{1}{3}}\right\}=n\ln t+n\ln K$$

where *n* and *K* are constant. The rate-limiting step is determined as diffusion when *n* = 0.5, while it is an interfacial reaction when *n* = 1. We applied this model to the swelling of LaNi_5_ powder as well as palladium [16]. ln{1 − (1 − *U*)^1/3^} at *U* < 0.8 is plotted with respect to ln(*t* − *t*_0_) in Figure 4, where *t*_0_ is the time at the maximum rate. ln{1 − (1 − *U*)^1/3^} increased with ln(*t* − *t*_0_), and its slope approaches almost 0.5. The slope *n* at 0.5 < *U* < 0.8 is 0.55–0.65 and 0.41–0.49 during the absorption and discharge, respectively. The deviation of *n* from 0.5 is due to the contribution of other reactions. 

The maximum rate proportional to the difference between *P_up_* and phase transition pressure leads to the rate-limiting step of Langmuir adsorption or Knudsen flow in voids according to [27]. However, the analysis does not take into account the effects of temperature change, stress gradient [28,29,30], and dislocations [31,32]. In particular, the phase transition to LaNi_5_H_6_ is an exothermal reaction, which changes the reaction rates. A more detailed investigation is required to clarify the contributions of these effects.

### 2.3. Suitability of LaNi_5_ as an Actuator Material

It is important to select a suitable material for an HSA actuator according to its application in order to enhance its performance. In this section, the suitability of LaNi_5_ as a material for an actuator is discussed through a comparison with palladium. Assume the actuator is driven by changing hydrogen pressure in the range of 0.0 and 0.5 MPa, and the applied load is negligibly small. Table 2 shows a summary of the performance under the assumed condition, where the result of palladium is shown in [16]. 

The swelling ratio divided by packing fraction *ε*/*f_V_* is 1.8 times larger in LaNi_5_ than palladium, which results in larger displacement as an actuator. Most of the swelling is achieved at the phase transition pressure. Therefore, the phase transition pressure should be included in the driving pressure range to obtain the large displacement of a actuator. For example, an actuator with LaNi_5_ can be driven at the upper pressure of >0.3 MPa and lower pressure of <0.1 MPa. 

The phase transition pressure also affects its response rate. The response time of LaNi_5_ at the time of the evacuation was almost double of that at the time of the pressurization, while 4000 times longer in palladium. As mentioned previously, the driving force of the reaction is dependent on the difference between the applied and equilibrium pressures. The equilibrium pressure corresponds to the phase transition pressure. The pressure difference |*P_ap_* − *P_e_*| of LaNi_5_ is ~0.3 and ~0.2 MPa during the absorption and discharge, respectively. In palladium, |*P_ap_* − *P_e_*| < 0.002 MPa during the discharge while |*P_ap_* − *P_e_*| = ~0.5 MPa during the absorption. This is why the palladium showed a large difference between the response times during the absorption and discharge although it has a large diffusion coefficient and the short response time during the absorption compared to LaNi_5_. Therefore, palladium is suitable for one-way quick operation such as a pressure relief valve for hydrogen. LaNi_5_ can realize a repeatable actuation under operation pressure at 0.5 MPa. 

As Kagawa [4] pointed out, the stability of the material is also important such as the pulverization and oxidation resistance. The pulverization does not occur in palladium thanks to large plastic deformation [33]. Unfortunately, LaNi_5_ shows the pulverization as observed the reduction of the mean volume diameter to approximately 10 μm in the present work. The dispersion of LaNi_5_ powder into polyurethane resin is effective to overcome the pulverization problem as shown in Okawa et al. [9] and Nishi et al. [10], though the displacement becomes smaller compared with pure LaNi_5_ film. This is a disadvantage of LaNi_5_. The cost is also critical from the viewpoint of economy. Palladium is “prohibitively [34]” expensive, and LaNi_5_ is more economical. Moreover, though the sample is subjected to less load in this work, hydrogen storage ability is changed by stress [28,29,30]. The swelling behavior change under stress is important information because an actuator receives the reactive force, which is a topic for future research.

## 3. Experiment

The displacement of LaNi_5_ powder (Japan Metals & Chemicals Co., Ltd., Tokyo, Japan) in hydrogen was measured using the same measurement system in [16]. The powder was packed into a cylinder made of aluminum alloy with an inner diameter of 13 mm, and a piston was put on the powder. The friction between the piston and cylinder was decreased by the vacuum grease (HIVAC-C, Shin-Etsu Chemical Co., Ltd., Tokyo, Japan). The cylinder was placed into a pressure chamber made of stainless. It has a viewing window made of silica glass, which makes it possible to measure the displacement at the top of the piston *u*, corresponding to the displacement of the powder, using a laser displacement sensor (LK-G85, Keyence Co., Osaka, Japan) through the window. The chamber was connected to a tank including hydrogen gas with a purity of 99.999% or more (Taiyo Nippon Sanyo Co., Tokyo, Japan) and a vacuum pump. It is noted that there is no pressure difference between inside and outside the cylinder as the gas is charged into the pressure chamber. 

**Figure 5:**
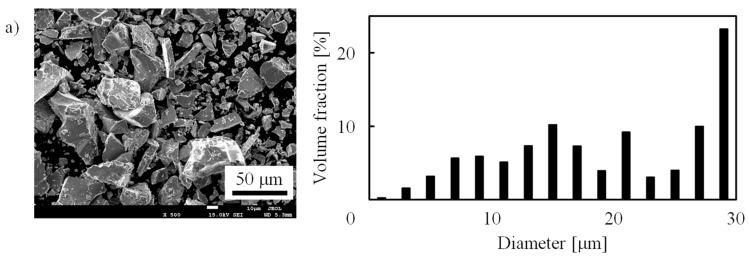

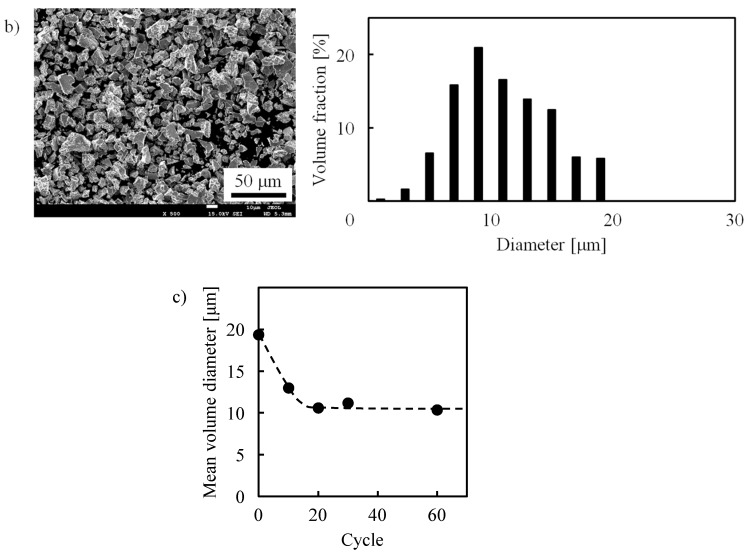
SEM images and size distributions of LaNi_5_ particles after (**a**) 0 and (**b**) 30 cycles of hydrogen charge, and (**c**) variation of mean volume diameter with cycle.

For an activation treatment, a hydrogen charge at ~0.7 MPa and the following evacuation were repeated 50 times before the measurements. Figure 5 shows SEM images and the particle size distributions of LaNi_5_ powder as-purchased (Figure 5a) and after 30 cycles of the hydrogen charge (Figure 5b). The particle sizes were measured from SEM images with an image processing software, ImageJ [35], and the mean volume diameter was calculated from more than 1000 particles. The particles were pulverized below 15 μm by hydrogen charge. The mean volume diameter was 11.1 μm after 30 cycles, which was almost constant after 20 cycles (Figure 5c). This value agrees with the previous studies [17]. 

The variation of the swelling ratio *ε* with equilibrium pressure *P_eq_* was measured by changing the pressure stepwise below 0.698 MPa. The height *h* and weight *w* of the sample were 1.37 mm and 1.00 g, respectively; the packing fraction *f_V_* was 0.67. The background due to the hydrostatic pressure was measured with changing the pressure under helium gas; the displacement increased in proportion to the helium pressure, i.e., 5.3 μm/MPa. An effect of the refractive index difference in hydrogen and helium is negligibly small, which was calculated based on Snell’s law. The displacement *u_measured_* under hydrogen was recorded after stabilization when it became constant, and, then, the background was subtracted according to *u* = *u_measured_* − 5.3*P_eq_*, where *u* is the true displacement. The swelling ratio *ε* was calculated as follows.

(2) $$\varepsilon =\frac{u}{h}$$

Hydrogen content change with the pressure was also obtained at room temperature with a Sievert’s type apparatus [36]. Note that no temperature regulation was attempted other than with an air conditioner. 

The swelling response time was measured by changing the pressure rapidly. At the kinetic measurement, *f_V_* = 0.59, where *h* = 1.61 mm and *w* = 1.03 g. Hydrogen was introduced into the evacuated chamber up to the target pressure *P_up_* for the investigation of swelling during absorption. The pressure reached 80% of *P_up_* within 4 s. For the discharge process, the chamber was evacuated from *P_up_* using the rotary vacuum pump. The recording interval was 1 s. 

## 4. Conclusions

In this work, the suitability of LaNi_5_ alloy as a material for an HSA actuator was discussed. The static and kinetic displacement changes with hydrogen pressure were investigated using LaNi_5_ powder. The results showed that the swelling ratio was 0.17 when 0.698 MPa of hydrogen was charged. In particular, it increased by 0.12 at 0.130–0.155 MPa, which agrees with the phase transition pressure. During the discharge, the equilibrium pressure was lower than that during the absorption at the same swelling ratio. The response time decreased with increase in the charged pressure during the absorption because the driving force of hydrogen absorption increased with the pressure. During the discharge, the response time and the driving force of hydrogen discharge were constant. The rate-limiting step of the swelling was diffusion in the latter part of the reaction. 

From the comparison of the swelling ability of LaNi_5_ with that of palladium, it was found that LaNi_5_ swells 1.8 times more than palladium under 0.5 MPa of hydrogen. LaNi_5_ showed a similar response time during the absorption and discharge (33 and 55 s, respectively), while the response time of palladium was 4000 times longer during the discharge than during the absorption. In conclusion, LaNi_5_ is suitable for an actuator driven repeatedly under more than 0.155 MPa. Palladium can be used for one-way-operation actuator even under 0.1 MPa. 

## Figures and Tables

**Table 1:** Material parameters of LaNi_5_ and palladium.

	LaNi_5_	Palladium
Phase transition pressure [MPa]		
During absorption	0.20 [20]	0.002 [16]
During discharge	0.16 [20]	0.0009 [16]
Lattice expansion ratio of hydride [−]	0.27 [22]	0.11 [23]
Diffusion coefficient of hydride at 300 K [m^2^/s]	1.5 × 10^−12^ [24]	1.3 × 10^−11^ [16]

**Table 2:** Performance of LaNi_5_ and palladium as an actuator when *P_up_* = 0.5 MPa.

	LaNi_5_	Palladium [16]
Swelling ratio/packing fraction *ε*/*f_V_* [−]	0.23	0.13
Response time at *U* = 0.8 *t*_80_ [s]		
During absorption	33	3
During discharge	55	12,230
Maximum response rate [s^−1^] (time [s])		
During absorption	0.041 (12)	0.46 (1.3)
During discharge	−0.013 (56)	−0.01 (2)

**Figure A1:**
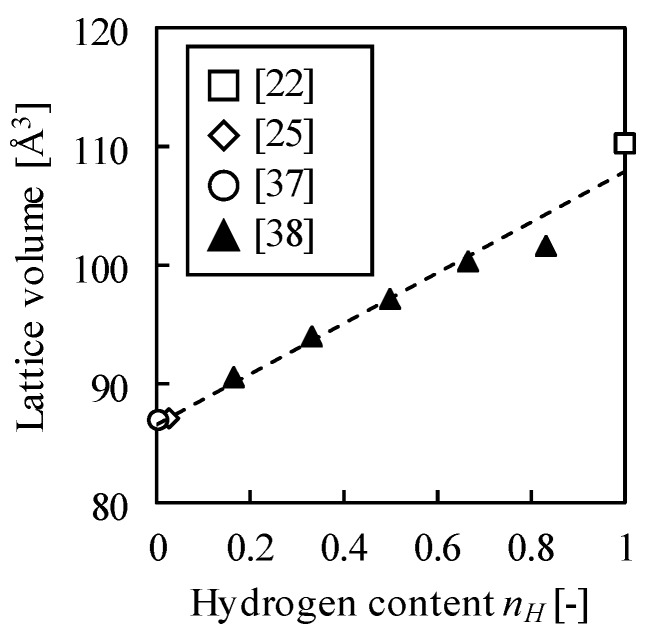
Dependence of lattice volume on hydrogen content.

## Appendix A. Dependence of Lattice Swelling Ratio on Hydrogen Content

Figure A1 shows the relationship between lattice volume and hydrogen content investigated experimentally [22,25,37] and numerically [38]. The lattice volume increases in proportion to the hydrogen content.
